# Research on a Lamb Wave and Particle Filter-Based On-Line Crack Propagation Prognosis Method

**DOI:** 10.3390/s16030320

**Published:** 2016-03-03

**Authors:** Jian Chen, Shenfang Yuan, Lei Qiu, Jian Cai, Weibo Yang

**Affiliations:** State Key Laboratory of Mechanics and Control of Mechanical Structures, Nanjing University of Aeronautics and Astronautics, 29 Yudao Street, Nanjing 210016, China; cj1108@nuaa.edu.cn (J.C.); lei.qiu@nuaa.edu.cn (L.Q.); caijian@nuaa.edu.cn (J.C.); ywb1987@nuaa.edu.cn (W.Y.)

**Keywords:** prognostics and health management, fatigue crack propagation, on-line, active Lamb wave monitoring, particle filter

## Abstract

Prognostics and health management techniques have drawn widespread attention due to their ability to facilitate maintenance activities based on need. On-line prognosis of fatigue crack propagation can offer information for optimizing operation and maintenance strategies in real-time. This paper proposes a Lamb wave-particle filter (LW-PF)-based method for on-line prognosis of fatigue crack propagation which takes advantages of the possibility of on-line monitoring to evaluate the actual crack length and uses a particle filter to deal with the crack evolution and monitoring uncertainties. The piezoelectric transducers (PZTs)-based active Lamb wave method is adopted for on-line crack monitoring. The state space model relating to crack propagation is established by the data-driven and finite element methods. Fatigue experiments performed on hole-edge crack specimens have validated the advantages of the proposed method.

## 1. Introduction

Reliability and availability are key problems for safety-critical systems such as aircraft, wind turbines, bridges and nuclear plants [1,2]. However, conventional maintenance frameworks may involve longer downtime and greater cost without considering the actual status of the systems [3]. In recent years, prognostics and health management (PHM) techniques, which consider the actual system condition via diagnostic techniques and the future condition through prognosis methods, have drawn widespread attention due to their ability to enable maintenance activities based on need [4,5,6]. Accurate prognosis for the degradation state and failure time of the critical structure plays an important role in the PHM technique, which leads to an increase of reliability and availability. Moreover, the life cycle cost will be reduced by undertaking maintenance activities only as necessary and minimizing downtime and spare part storage.

Fatigue cracks are commonly regarded as a principal failure mode for various structural and mechanical systems [7,8]. In recent years, a lot of attention has been paid to the methods which combine fatigue crack propagation models with Bayes’ theorem for fatigue crack propagation prognosis, including stochastic filters [9,10,11,12,13,14,15] and the Bayesian inference [16,17,18]. Within these methods, the uncertainties during fatigue crack propagation are taken into account. The measurement information of the actual crack propagation state is used to update the result obtained by the crack propagation model to achieve a more accurate one. As to stochastic filters, the Kalman filter (KF) [19] offers the optimal solution to linear problems under Gaussian uncertainty assumption. Nevertheless, most realistic cases such as the process of fatigue crack propagation are nonlinear with non-Gaussian uncertainty. To tackle these cases, various types of approximation are developed for the KF [20]. The extended Kalman filter (EKF) utilizes the first-order Taylor expansion of the nonlinear model to solve the fatigue crack propagation problem [9]. The unscented Kalman filter (UKF) is proposed to deal with the fatigue crack propagation problem using unscented transformation [10]. However, the Gaussian uncertainty assumption is still needed for the EKF and UKF. As an improvement of the KF, the particle filter (PF) is capable of handling the prognosis problem of nonlinear and non-Gaussian processes without restrictive assumptions based on Monte Carlo methods [21]. In recent years, several PF based methods have been reported for fatigue crack propagation prognosis. Shin *et al.* [11] adopted the PF to deal with the prognosis problem of fatigue crack propagation. The visual inspections performed by an optical magnifier were used as the measurements of the crack lengths. Corbetta *et al.* [12] proposed a kind of stochastic dynamic state space model for the PF based method, which utilized the crack lengths obtained with a caliper as the measurements. Compare and Zio [13] proposed a PF-based method for predictive maintenance, in which simulated crack lengths were employed for validation. Sun *et al.* [14] analyzed the sources of uncertainties in fatigue crack propagation prognosis using Virkler’s data which was measured by a zoom stereomicroscope and explored a PF-based algorithm for uncertainty management. All these studies indicate that the PF has the potential for fatigue crack propagation prognosis under uncertainties.

However, most literatures published so far have only used simulation or off-line NDT results, which have limitations for on-line application. On-line crack monitoring is capable of offering convenient and quick inspections of crack damages with sensors. Timely detection and prognosis of crack damages can maximize the operational availability and safety by optimizing operation and maintenance strategies in real-time. Recently, on-line crack monitoring methods have been gradually combined with PF-based methods to realize on-line crack propagation prognosis. For example, Chen *et al.* [15] proposed a PF-based method for the machine condition prediction, in which the vibration feature extraction method is applied for crack monitoring. However, vibration feature extraction- based methods are insensitive to small damage or damage growth [22]. In general, research on prognosis methods integrating on-line crack monitoring with the PF is still lacking, as well as experimental verifications. With the development of the structural health monitoring (SHM) technology, different kinds of methods have been developed for on-line crack monitoring [23]. Among them, The PZTs-based active Lamb wave (LW) technique is one of the most appealing and effective methods [24,25,26], which has the merits including the ability of traveling a long distance, the capacity to access hidden components, as well as sensitivity to small crack damages [27,28].

Aiming at realizing on-line fatigue crack propagation prognosis, a LW-PF-based method is proposed to combine the PZTs-based active Lamb wave method with the PF. The PZT sensor array is used to actuate and acquire Lamb wave signals in the structure. The cross-correlation damage index extracted from the monitored Lamb wave signal is employed to capture signal characteristics and quantify the actual crack length. Each time a new damage index is available, the PF utilizes this damage index to estimate the posterior probability density function (pdf) of the crack length with the crack propagation state space model. This state space model is derived from a stochastic Paris’s law and an active Lamb wave-based measurement equation, whose parameters and uncertainty are determined by data driven methods. On the basis of the obtained posterior pdf, the prognosis of the crack propagation is performed and the failure cycle is calculated afterwards. The proposed method is evaluated with the fatigue experiments of 6 hole-edge crack specimens, and the posterior estimation and prognostic value of the crack length are discussed, as well as the failure cycle.

The rest of the paper is organized as follows: in Section 2, the proposed on-line LW-PF-based crack propagation prognosis method is explained. First, the state equation of the fatigue crack propagation is presented. Then, the PZTs-based active Lamb wave method is introduced. The modeling process of the measurement equation is proposed with the cross-correlation damage indices extracted from experimental Lamb wave signals. At the end, the LW-PF based prognosis method is presented in detail. In Section 3, the proposed method is implemented and validated on 6 hole-edge crack specimens. Finally, the conclusions are given in Section 4.

## 2. On-Line LW-PF-Based Crack Propagation Prognosis Method

To apply the PF-based method, essentially a state space model describing fatigue crack propagation is needed, which consists of a state equation and a measurement equation. The state equation describes the evolution of the crack length, and the measurement equation governs the relationship between the damage index and the crack length.

### 2.1. Physical Model Based State Equation with Data Driven Parameters

In this section, the method of establishing the state equation is proposed, in which the crack propagation mechanism is based on Paris’ law with the parameters obtained by data driven methods.

#### 2.1.1. Stochastic Paris’ Law Based State Equation

The general state equation of fatigue crack propagation can be expressed by Equation (1):

(1) $$x_{k}=f(x_{k-1},\omega_{k-1})$$

where *k* is the discrete time index, $x_{k}$ is the crack length at time *k*, f(⋅) is a nonlinear function representing the evolution of the crack length from time *k* − 1 to time *k*, $\omega_{k-1}$ is a random variable denoting the uncertainty during fatigue crack propagation.

Paris’ law [29] has been widely used to describe fatigue crack propagation, giving the crack propagation rate as Equation (2):

(2) $$\frac{dx}{dN}=C{\left(\Delta K\right)}^{m}$$

where *N* is the number of loading cycles, *C* and *m* are material constants, ΔK is the stress intensity factor (SIF) range determined by the crack length and the fatigue load.

To take the uncertainty during fatigue crack propagation into consideration, the crack propagation rate can be modified as Equation (3) according to Yang and Manning’s model [30]:

(3) $$\frac{dx}{dN}=X(N)C{\left(\Delta K\right)}^{m}$$

where *X*(*N*) is a stationary random process denoting the uncertainty during fatigue crack propagation. For simplification, *X*(*N*) can be reduced to a lognormal random variable *X* [31] and expressed as exp(ω), where ω follows the normal distribution $N(0,\sigma_{\omega }^{2})$. As a result, Equation (3) can be discretized as the state Equation (4):

(4) $$x_{k}=x_{k-1}+\exp(\omega )C{\left(\Delta K\right)}^{m}\Delta N$$

where ΔN is the discrete step of loading cycles.

#### 2.1.2. Parameters of the State Equation

**Figure 1:**
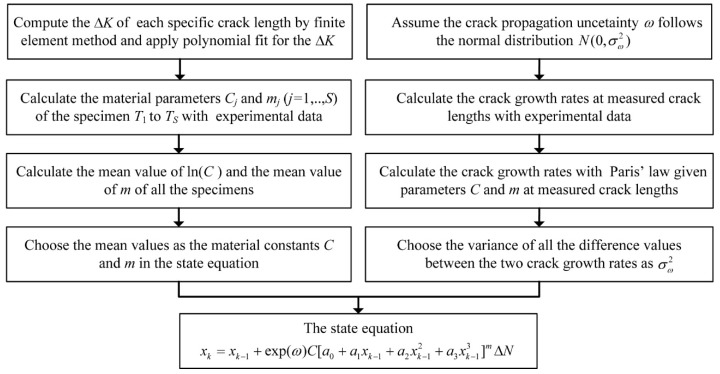
The process to obtain the state equation parameters.

The parameters Δ*K*, *C*, *m*, and $\sigma_{\omega }^{2}$ in Equation (4) should be determined in advance for prognosis. Since different kinds of structures have different parameters, the parameters of a certain kind of structure need to be decided specifically. A data driven method is proposed to obtain the parameters as the process shown in Figure 1, where the variable *S* denotes the number of the specimens.

##### Computation of the Δ*K* with the Finite Element Method

First, the finite element method (FEM) is adopted to compute the SIF range Δ*K*. Given the structure, the crack with specific crack length and position can be simulated in the finite element model by introducing discontinuities between elements.

The Δ*K* is then calculated through *J*-integral after applying the boundary conditions and the load range $\Delta P=P_{\max}-P_{\min}$. To improve the numerical results, singular elements and fine mesh are applied around the crack tip [32]. By repeating analyses for cracks with different lengths assuming the crack position is constant, a set of SIF ranges can be acquired. A third-order polynomial approximation is then applied for the relationship between the crack length and Δ*K* as expressed in Equation (5):

(5) $$\Delta K(x)=a_{0}+a_{1}x+a_{2}x^{2}+a_{3}x^{3}$$

where $\left\{a_{n},\;n=0,1,2,3\right\}$ are the polynomial coefficients, *x* is the crack length.

##### Calculating Material Constants

Fatigue experiments are performed for a set of this kind of structures to determine the material constants *C* and *m*. The logarithmic transformation of Equation (2) is expressed as Equation (6):

(6) $$\ln(\frac{dx}{dN})=\ln(C)+m\ln(\Delta K)$$

where ln(*C*) and *m* are the intercept and slope considering the linear relationship between ln(dx/dN) and ln(ΔK).

During the experiments, *S* specimens labeled as $\left\{T_{j},j=1,\;\ldots ,\;S\right\}$ are tested and measurements of the crack lengths $\left\{x_{i}^{j},\;i=1,\;...,M\right\}$ are performed *M* times for each specimen. Their corresponding loading cycles are recorded as $\left\{N_{i}^{j},\;i=1,\;...,M\right\}$. It should be noted that $x_{0}^{j}$ is the crack length first measured, and $N_{0}^{j}$ is the corresponding loading cycle. The crack propagation rate at crack length $x_{i}^{j}$ of the specimen *T_j_* can be calculated approximately using the experimental data as shown in Equation (7):

(7) $${\left(\frac{dx}{dN}\right)}_{i}^{j}\approx \frac{\Delta x_{i}^{j}}{\Delta N_{i}^{j}}=\frac{x_{i}^{j}-x_{i-1}^{j}}{N_{i}^{j}-N_{i-1}^{j}}$$

Taking these crack propagation rates $\left\{{\left(dx/dN\right)}_{i}^{j},\;i=1,...,M\right\}$ of each specimen with corresponding SIF ranges $\left\{\Delta K(x_{i}^{j}),\;i=1,....,M\right\}$, the two parameters $\ln(C_{j})$ and $m_{j}$ of the specimen $T_{j}$ are calculated using linear regression [31]. In this paper, the mean value of $\ln(C_{j})$ and the mean value of $m_{j}$ are adopted as the material constants in the state equation.

##### Estimation of the Crack Propagation Uncertainty

The logarithm transformation of Equation (4) is shown as Equation (8). Empirically, the difference between the logarithm of the crack propagation rate obtained from the experimental observations and the one from Paris’ law is used to approximate the crack propagation uncertainty as illustrated in Equation (9):

(8) $$\ln(\frac{\Delta x}{\Delta N})=\ln[C{\left(\Delta K\right)}^{m}]+\omega$$

(9) $$e_{i}^{j}=\ln(\frac{\Delta x_{i}^{j}}{\Delta N_{i}^{j}})-\ln\{C{\left[\Delta K(x_{i}^{j})\right]}^{m}\}$$

where the material constants *C* and *m* are determined previously, the difference value $e_{i}^{j}$ is supposed to be the crack propagation uncertainty that disturbs the crack propagation rate. Therefore, the variance $\sigma_{\omega }^{2}$ of the random variable ω is calculated as the variance of all the difference values from all the specimens as shown in Equation (10):

(10) $$\sigma_{\omega }^{2}\approx \text{Var}\left\{e_{i}^{j},\;i=1,...,M,\;j=1,...,S\right\}$$

where Var denotes the variance of the difference values.

After these processes, the state equation is obtained as Equation (11):

(11) $$x_{k}=x_{k-1}+\exp(\omega )C{\left[a_{0}+a_{1}x_{k-1}+a_{2}x_{k-1}^{2}+a_{3}x_{k-1}^{3}\right]}^{m}\Delta N$$

### 2.2. Active Lamb Wave-Based Measurement Equation

#### 2.2.1. On-Line Crack Monitoring by the Active Lamb Wave Method

**Figure 2:**
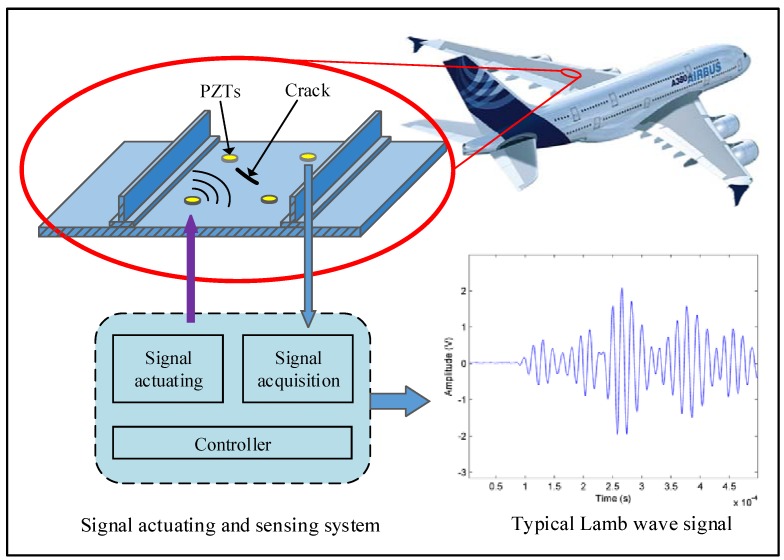
The PZTs-based active Lamb wave method for on-line crack monitoring.

The PZTs-based active Lamb wave method is adopted for on-line crack monitoring. Lamb waves are elastic waves that propagate in plate-like structures. A typical configuration used for crack monitoring is the pitch-catch way as shown in Figure 2. 

The PZT sensor array is arranged on the structure. After the Lamb wave is excited in the structure by a PZT, interaction of the Lamb wave with the crack can influence the Lamb wave propagation. By comparing the received signals under healthy and cracked conditions from other PZTs, the crack can be estimated.

To evaluate the variations between the baseline signal collected when the structure is healthy and the monitored signal when the crack propagates in the structure, many damage indices have been proposed [22]. In this paper, the cross-correlation damage index is adopted to quantify the crack length as expressed in Equation (12):

(12) $$DI=1-\sqrt{\frac{{\left\{\int_{t_{0}}^{t_{1}}H\left(t\right)D\left(t\right)dt\right\}}^{2}}{\left\{\int_{t_{0}}^{t_{1}}H^{2}\left(t\right)dt\int_{t_{0}}^{t_{1}}D^{2}\left(t\right)dt\right\}}}$$

where *H*(*t*) is the baseline signal, *D*(*t*) is the monitored signal during the crack propagation process, *t*_0_ and *t*_1_ are the sampling start time and stop time. This damage index reflects the phase changes between the signals caused by the increasing crack length.

#### 2.2.2. Active Lamb Wave-Based Measurement Equation

The measurement equation is defined as Equation (13):

(13) $$y_{k}=g(x_{k},\nu )$$

where the function g(⋅) represents the relationship between the damage index $y_{k}$ and the crack length $x_{k}$, ν is a random variable denoting the measurement uncertainty.

**Figure 3:**
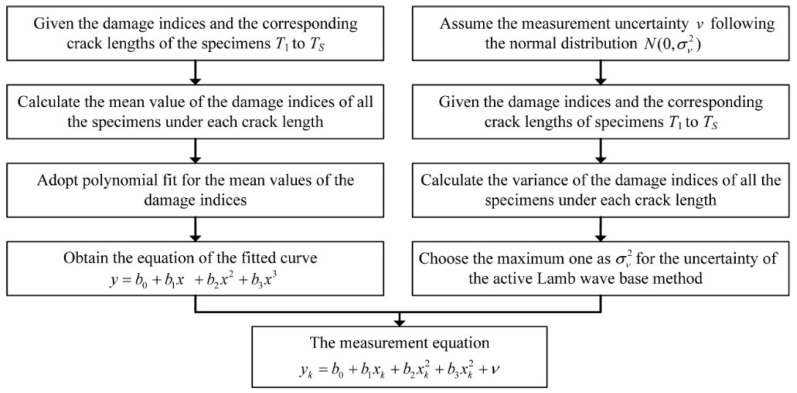
The process to establish the measurement equation by the data driven method.

The proposed active Lamb wave-based measurement equation is established by the data driven method as shown in Figure 3.

During the fatigue experiment of each specimen $T_{j},\;j=1,...,S$, the baseline signal is acquired from the initial state. With the crack propagation in the specimen $T_{j}$, damage indices are obtained at *M* specific crack lengths $\left\{x_{i},\;i=1,...,M\right\}$, which are denoted as $\left\{y_{i}^{j},\;i=1,...,M\right\}$. The mean value of the damage indices at the crack length $x_{i}$ of all the specimens can be calculated as Equation (14):

(14) $${\bar{y}}_{i}=\frac{\sum_{j=1}^{S}y_{i}^{j}}{S}$$

After that, a third order polynomial as shown in Equation (15) is employed to govern the relationship between the crack length and the mean value of the damage indices:

(15) $$y=b_{0}+b_{1}x+b_{2}x^{2}+b_{3}x^{3}$$

where $\left\{b_{n},n=0,1,2,3\right\}$ are the polynomial coefficients.

The measurement uncertainty is assumed to be normally distributed, which denotes $\nu ∼N(0,\sigma_{\nu }^{2})$. With the damage indices obtained from all the specimens, the variance ${\left(\sigma_{v}^{2}\right)}_{i}$ of the damage indices at crack length $x_{i}$ can be approximated as Equation (16):

(16) $${\left(\sigma_{v}^{2}\right)}_{i}\approx \text{Var}\left\{y_{i}^{j},\;j=1,...,S\right\}$$

where Var is the variance of the damage indices.

As a result, the maximum one in the set $\left\{{\left(\sigma_{v}^{2}\right)}_{i},\;i=1,...,M\right\}$ is adopted as $\sigma_{v}^{2}$ of the measurement uncertainty to illustrate the capability the proposed method.

Hence, the measurement equation is obtained as Equation (17):

(17) $$y_{k}=b_{0}+b_{1}x_{k}+b_{2}x_{k}^{2}+b_{3}x_{k}^{2}+\nu$$

### 2.3. On-Line LW-PF-Based Crack Propagation Prognosis Method

Assuming the current time is *k*, the objective of the prognosis method is to predict the crack length $x_{k+d}$, where *k* + *d* is the future time of interest. In this paper, the PF is employed to incorporate the damage index of the actual crack state for a more precise prognostic result, which gives the conditional pdf $p(x_{k+d}|y_{1:k})$ with the on-line monitored damage indices $y_{1:k}=\{y_{j},j=1,...,k\}$. This conditional pdf represents the probability distribution of $x_{k+d}$ and contains all the available measurement information to the current time *k*. Then the estimation of the crack length ${\hat{x}}_{k+d}$ can be expressed as Equation (18):

(18) $${\hat{x}}_{k+d}=\int x_{k+d}p(x_{k+d}|y_{1:k})dx_{k+d}$$

It should be noted that the latest damage index in $p(x_{k+d}|y_{1:k})$ can be obtained at time *k* is $y_{k}$. There are two procedures in the proposed LW-PF method: the first one is to estimate the posterior pdf $p(x_{k}|y_{1:k})$ of the current crack length $x_{k}$ according to the on-line monitored damage indices $y_{1:k}$. The second procedure is to predict the future crack length $x_{k+d}$ on the basis of the posterior pdf $p(x_{k}|y_{1:k})$.

#### 2.3.1. Estimation of the Posterior Pdf with On-Line Monitored Damage Indices

From a Bayesian perspective, the posterior pdf $p(x_{k}|y_{1:k})$ of the crack length $x_{k}$ is recursively calculated with on-line monitored damage indices in two stages: prediction and update.

Suppose the posterior pdf $p(x_{k-1}|y_{1:k-1})$ at time *k* − 1 is obtained. The prior pdf $p(x_{k}|y_{1:k-1})$ can be calculated by Equation (19) in the prediction step, where $p(x_{k}|x_{k-1})$ is the transition pdf defined by Equation (11). Once the damage index $y_{k}$ is obtained, it is used to calculate the posterior pdf $p(x_{k}|y_{1:k})$ through Bayes’ theorem as expressed in Equation (20):

(19) $$\text{Prediction}:\;p(x_{k}|y_{1:k-1})=\int p(x_{k}|x_{k-1})p(x_{k-1}|y_{1:k-1})dx_{k-1}$$

(20) $$\text{Update}:\;p(x_{k}|y_{1:k})=\frac{p(y_{k}|x_{k})p(x_{k}|y_{1:k-1})}{p(y_{k}|y_{1:k-1})}$$

where $p(y_{k}|x_{k})$ is the likelihood function defined by the measurement equation, $p(y_{k}|y_{1:k-1})$ is a normalized constant [21].

If the posterior pdf $p(x_{k}|y_{1:k})$ is obtained, the posterior estimation of the crack length $x_{k}$ can be calculated as Equation (21):

(21) $${\hat{x}}_{k}=\int x_{k}p(x_{k}|y_{1:k})dx_{k}$$

However, Equations (19) and (20) do not have analytical solutions in the most cases. The PF makes the evaluation feasible by resorting to Monte Carlo methods. The basic idea of the PF is to approximate the posterior pdf $p(x_{k}|y_{1:k})$ by means of a set of particles $\left\{x_{k}^{\left(i\right)},i=1,...,N_{s}\right\}$ with their normalized weights $\left\{{\tilde{w}}_{k}^{\left(i\right)},i=1,...,N_{s}\right\}$:

(22) $$p(x_{k}|y_{1:k})\approx \sum_{i=1}^{N_{s}}{\tilde{w}}_{k}^{\left(i\right)}\delta (x_{k}-x_{k}^{\left(i\right)})$$

where δ is the Dirac delta function, $N_{s}$ is the number of particles, $\left\{x_{k}^{\left(i\right)},i=1,...,N_{s}\right\}$ are particles sampled from the pdf $p(x_{k}|y_{1:k})$. Since the posterior pdf is difficult to sampled from, instead, these particles are sampled from a known and easily sampled pdf $q(x_{k}|y_{1:k})$ called the importance density function [21]. Hence, the posterior estimation of $x_{k}$ can be expressed as Equation (23), and the corresponding weight $w_{k}^{\left(i\right)}$ is defined as Equation (24):

(23) $${\hat{x}}_{k}=\int x_{k}\frac{p(x_{k}|y_{1:k})}{q(x_{k}|y_{1:k})}q(x_{k}|y_{1:k})dx_{k}$$

(24) $$w_{k}^{\left(i\right)}\propto \frac{p(x_{k}^{\left(i\right)}|y_{1:k})}{q(x_{k}^{\left(i\right)}|y_{1:k})}$$

If the importance density function is chosen to factorize such that:

(25) $$q(x_{k}|y_{1:k})=q(x_{k-1}|y_{1:k-1})q(x_{k}|x_{k-1},y_{1:k})$$

the non-normalized weight $w_{k}^{\left(i\right)}$ can be derived in a iterative form as shown in Equation (26) [20], and the weight normalization is given as Equation (27):

(26) $$w_{k}^{\left(i\right)}=w_{k-1}^{\left(i\right)}\frac{p(y_{k}|x_{k}^{\left(i\right)})p(x_{k}^{\left(i\right)}|x_{k-1}^{\left(i\right)})}{q(x_{k}^{\left(i\right)}|y_{1:k})}$$

(27) $${\tilde{w}}_{k}^{\left(i\right)}=\frac{w_{k}^{\left(i\right)}}{\sum_{i=1}^{N}w_{k}^{\left(i\right)}}$$

where, $p(y_{k}|x_{k}^{\left(i\right)})$ is the likelihood value corresponding to the particle $x_{k}^{\left(i\right)}$ given the damage index $y_{k}$. Due to the normal distribution assumption of the measurement uncertainty, this likelihood value is calculated as Equation (28):

(28) $$p(y_{k}|x_{k}^{\left(i\right)})=\frac{1}{\sqrt{2\pi \sigma_{v}^{2}}}e^{-\frac{y_{k}-[b_{0}+b_{1}(x_{k}^{\left(i\right)})+b_{2}{\left(x_{k}^{\left(i\right)}\right)}^{2}+b_{3}{\left(x_{k}^{\left(i\right)}\right)}^{3}]}{2\sigma_{v}^{2}}}$$

A common choice of the importance density function is the transition pdf, *i.e.*, $q(x_{k}^{\left(i\right)}|y_{1:k})=p(x_{k}^{\left(i\right)}|x_{k-1}^{\left(i\right)})$. In this sense, the calculation of the weights can be reduced to Equation (29):

(29) $$w_{k}^{\left(i\right)}=w_{k-1}^{\left(i\right)}p(y_{k}|x_{k}^{\left(i\right)})$$

One of the main problems of this PF is the degeneracy phenomenon. After a few iterations, all but one particle will have negligible weight. The effective sample size $N_{eff}$ expressed in Equation (30) is introduced to evaluate the degradation degree [21]. To deal with this problem, a resampling procedure is performed when $N_{eff}$ is less than a fixed threshold $N_{th}$. The resampling eliminates particles with small weights, copying which have large weights, and setting all the weights to $1/N_{s}$:

(30) $$N_{eff}=\frac{N_{s}}{1+Var({\tilde{w}}_{k}^{\left(i\right)})}\approx \frac{1}{\sum_{i=1}^{N_{s}}{\left({\tilde{w}}_{k}^{\left(i\right)}\right)}^{2}}$$

**Figure 4:**
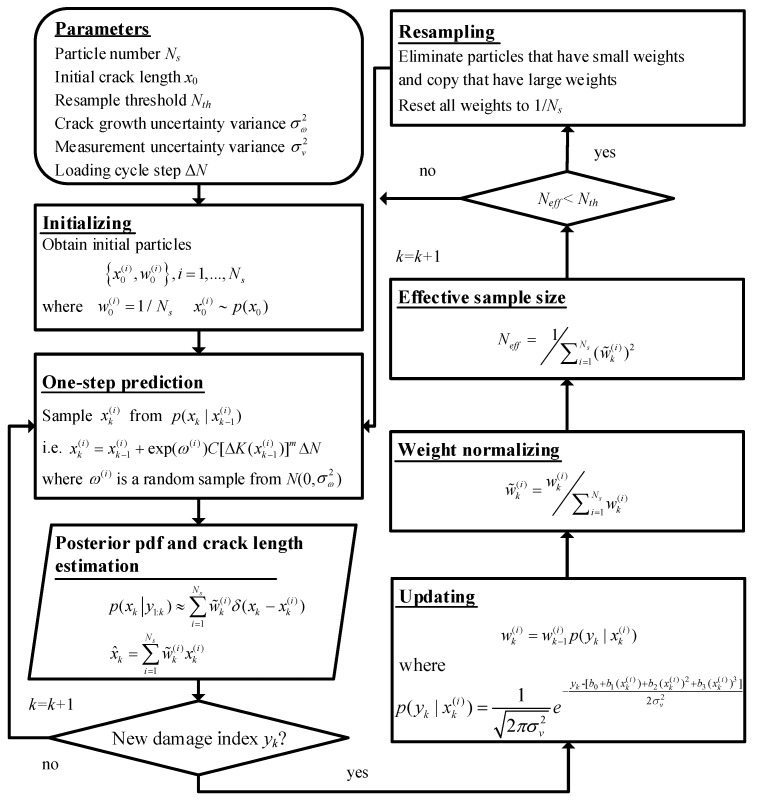
Flow chart for estimating the crack length posterior pdf with the LW-PF.

As a result, the flow chart to estimate the posterior pdf of the crack length is illustrated in Figure 4.

#### 2.3.2. On-Line Prognosis of Fatigue Crack Propagation

After the posterior pdf $p(x_{k}|y_{1:k})$ is obtained, the prognosis procedure is performed. During this procedure, weight updates are no longer taken since no new damage indices can be collected. The condition pdf $p(x_{k+d}|y_{1:k})$ is calculated with the posterior pdf $p(x_{k}|y_{1:k})$ under the hypothesis of the Markov processes of order one as shown in Equation (31):

(31) $$p(x_{k+d}|y_{1:k})=\int p(x_{k}|y_{1:k})\prod_{j=k+1}^{k+d}p(x_{j}|x_{j-1})dx_{k:k+d-1}$$

Replacing $p(x_{k}|y_{1:k})$ as its approximation with the particles and corresponding weights gives Equation (32):

(32) $$p(x_{k+d}|y_{1:k})\approx \sum_{i=1}^{N_{s}}{\tilde{w}}_{k}^{\left(i\right)}\int ...\int p(x_{k+1}|x_{k}^{\left(i\right)})\prod_{j=k+2}^{k+d}p(x_{j}|x_{j-1})dx_{k+1:k+d-1}$$

These integrals can be evaluated by extending the particle $x_{k}^{\left(i\right)}$ using the state equation as shown in Equation (33). The conditional probability $p(x_{k+d}|y_{1:k})$ is approximated by the particles $\left\{x_{k+d}^{\left(i\right)},i=1,...,N_{s}\right\}$ and the unchanged weights $\left\{{\tilde{w}}_{k}^{\left(i\right)},i=1,...,N_{s}\right\}$ as expressed in Equation (34):

(33) $$x_{k+d}^{\left(i\right)}=x_{k+d-1}^{\left(i\right)}+\exp(\omega )C{\left[\Delta K(x_{k+d-1}^{\left(i\right)})\right]}^{m}\Delta N$$

(34) $$p(x_{k+d}|y_{1:k})\approx \sum_{i=1}^{N_{s}}{\tilde{w}}_{k}^{\left(i\right)}\delta (x_{k+d}-x_{k+d}^{\left(i\right)})$$

After that, the prognostic result of the crack length ${\hat{x}}_{k+d}$ can be evaluated as Equation (35):

(35) $${\hat{x}}_{k+d}=\sum_{i=1}^{N_{s}}{\tilde{w}}_{k}^{\left(i\right)}x_{k+d}^{\left(i\right)}$$

#### 2.3.3. Estimation of the Failure Cycle for the Structure

As to the fatigue crack propagation problem, the failure time of the structure may be represented by the failure cycle $N_{f}$, which indicates the loading cycle when the cracked structure becomes unusable. Usually, a threshold crack length $x_{th}$ is defined to evaluate whether this structure is still usable. Once the crack length exceeds this threshold, the cracked structure is regarded as failed. As mentioned above, each particle with its weight represents a possible crack propagation path. The failure cycle is estimated by collecting the loading cycle when each path reaches the threshold $x_{th}$. The pdf of the failure cycle can be expressed as Equation (36):

(36) $$p(N_{f}|y_{1:k})\approx \sum_{i=1}^{N_{s}}{\tilde{w}}_{k}^{\left(i\right)}\delta (N_{f}-{N_{f}}^{\left(i\right)})$$

where $N_{f}^{\left(i\right)}$ is the failure cycle of the i th crack propagation path.

Then the failure cycle is evaluated as Equation (37):

(37) $${\hat{N}}_{f}=\sum_{i=1}^{N_{s}}{\tilde{w}}_{k}^{\left(i\right)}N_{f}^{\left(i\right)}$$

**Figure 5:**
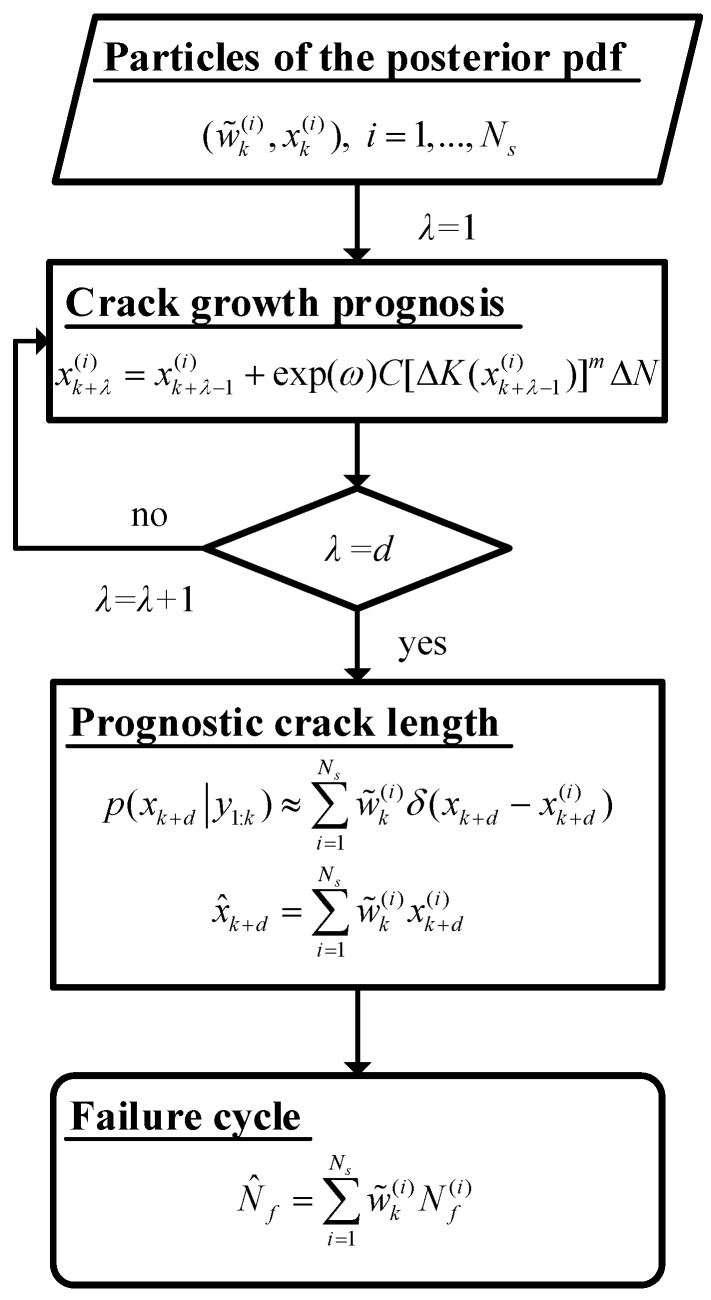
Flow chart of the prognosis for the crack propagation and failure cycle.

As illustrated in Figure 5, the flow chart shows the process to obtain the crack propagation prognosis and the estimation of the failure cycle based on the obtained posterior pdf.

## 3. Experimental Evaluation on Hole-Edge Crack Specimens

### 3.1. Experimental Setup

**Figure 6:**
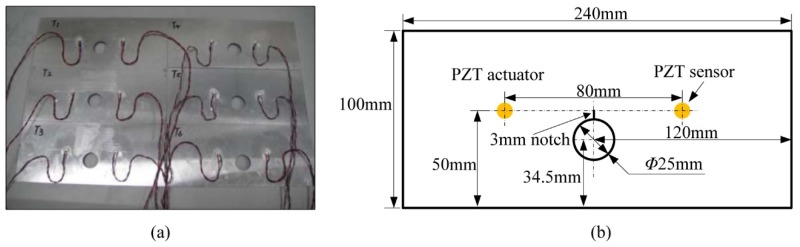
The hole-edge crack specimens: (**a**) The specimens; (**b**) The geometry and PZTs layout.

The experiments of 6 hole-edge crack specimens were performed to verify the effectiveness of the proposed method. The specimens were made of 2 mm thick YL12 aluminum plate and labeled from *T*_1_ to *T*_6_ as shown in Figure 6a. 

Table 1 lists their mechanical properties. For each specimen, a 3 mm notch was machined at the edge of the through hole to initiate the crack and control the crack propagation direction during the fatigue test. Two PZTs were attached to the specimen and used as the actuator and the sensor respectively, whose positions are illustrated in Figure 6b.

**Figure 7:**
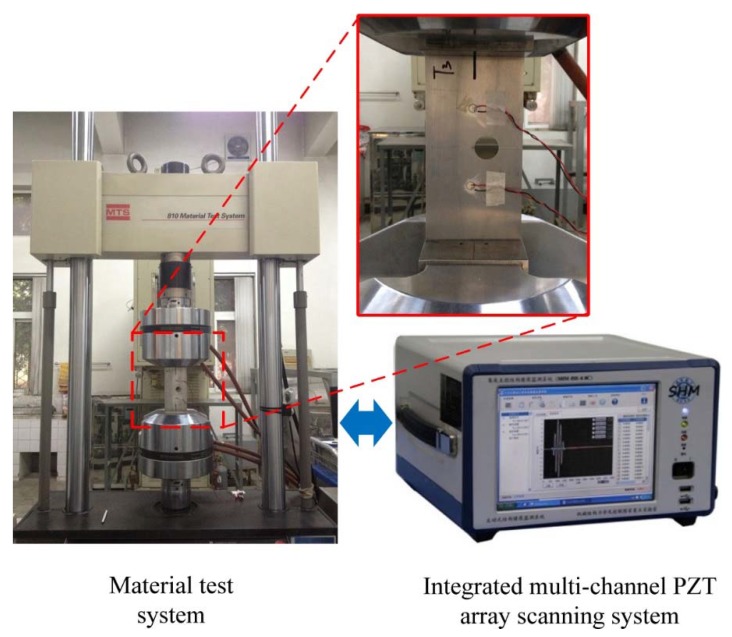
The experimental setup.

The experimental setup is illustrated in Figure 7. The material test system MTS810 was used for applying the fatigue load. The multi-channel PZT array scanning system developed by the authors’ group [33] was employed to perform the active Lamb wave based monitoring. A 5-cycle tone-burst signal with the center frequency of 290 kHz and ±10 V amplitude was used as the excitation signal, which is expressed in Equation (38). Lamb wave signals were sampled at 10 MHz:

(38) $$u\left(t\right)=A\left(1-\cos\frac{2\pi f_{c}t}{N_{c}}\right)\sin\left(2\pi f_{c}t\right)$$

where *N_c_* = 5, *f_c_* is the central frequency, *A* is the amplitude.

In this validation research, a tensile test was conducted at first for the specimen *T*_6_. The ultimate tensile load of the specimen *T*_6_ was obtained as 45 kN. Referring to this result, a sinusoidal load with peak value *P*_max_ = 15 kN was chosen for the fatigue experiments of the specimens *T*_1_ to *T*_5_, with the frequency of 10 Hz and the load ratio *R* = *P*_min_/*P*_max_ = 0.1.

### 3.2. Fatigue Crack Propagation and Damage Indices

**Figure 8:**
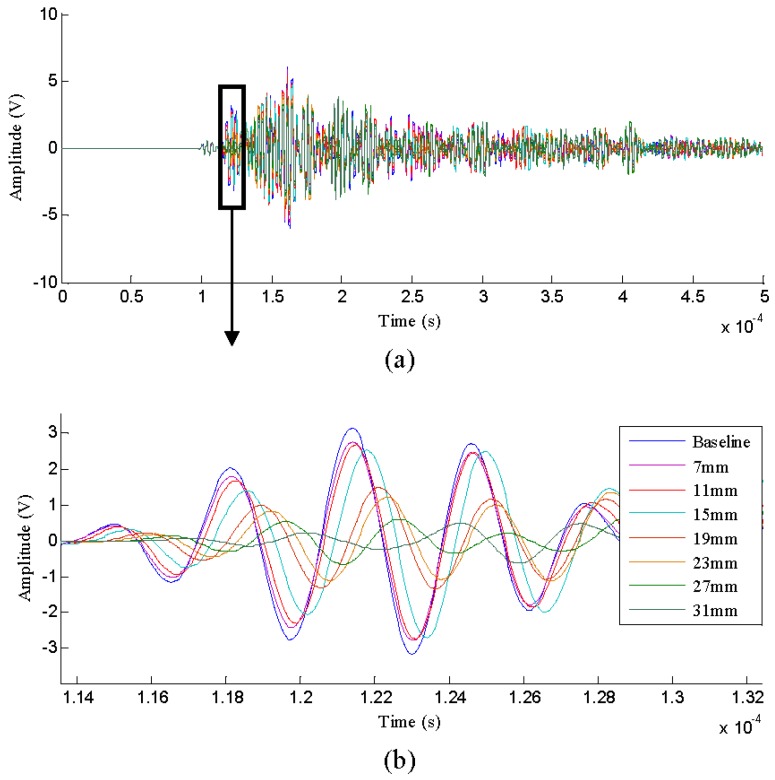
The typical Lamb wave signals of specimen *T*_1_: (**a**) Whole signal; (**b**) Local magnification.

The signal under the initial state of each specimen was collected as the baseline signal. Figure 8 illustrates a set of typical Lamb wave signals obtained as the fatigue crack propagation of the specimen *T*_1_. It is observed that the amplitudes and phases of the signals are influenced by the increasing crack lengths.

**Figure 9:**
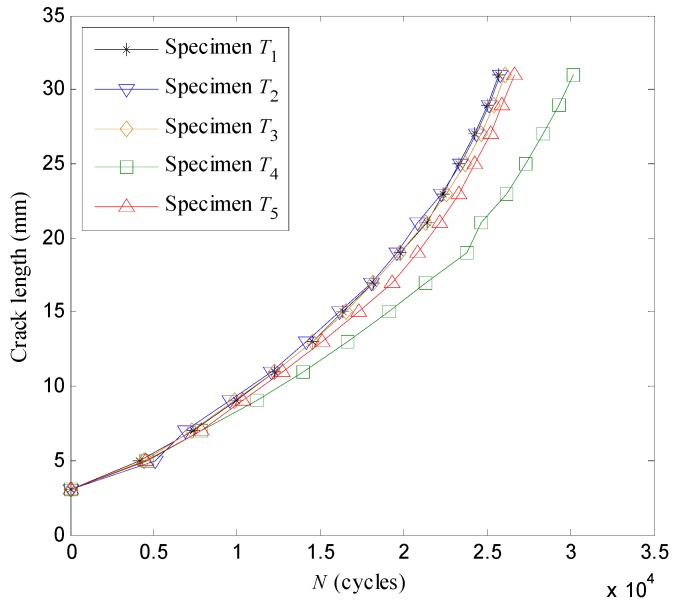
The crack lengths *versus* the loading cycles.

As illustrated in Figure 9, the fatigue crack propagation results of the specimens *T*_1_–*T*_5_ are recorded at 2 mm crack length intervals. 

**Figure 10:**
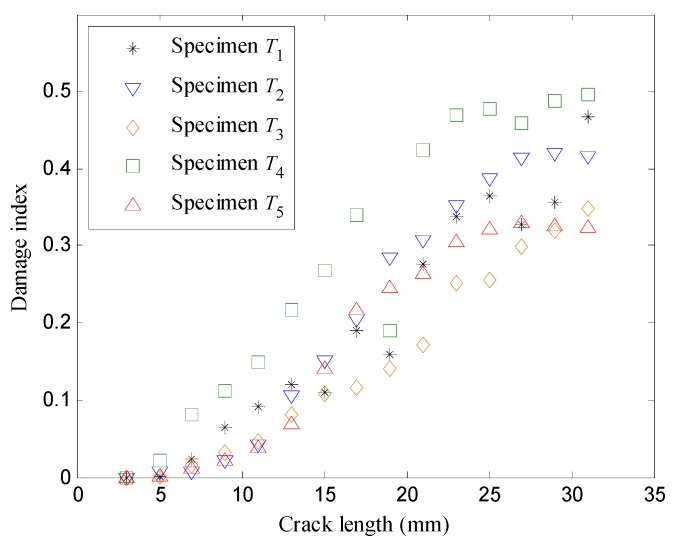
The damage indices *versus* the crack lengths.

Obvious uncertainties can be seen in the results. The corresponding damage indices are presented in Figure 10. It can be observed that the damage indices of each specimen are capable of representing the crack propagation process, and likewise measurement uncertainty exists.

### 3.3. On-Line Crack Propagation Estimation and Prognosis of the Specimen T_5_

In this validation research, the specimen *T*_5_ was used as the target structure and deemed as unknown. For the on-line crack propagation prognosis of the specimen *T*_5_, the information of the crack propagation of this kind of structure should be extracted to establish the state space model. Thus, the fatigue experiments of the specimens *T*_1_–*T*_4_ were conducted ahead to establish the state space model of the specimen *T*_5_.

#### 3.3.1. Establishing the State Space Model for the Specimen *T*_5_

As the process described in Section 2, the expression of ΔK was obtained as Equation (39):

(39) $$\Delta K=0.016x^{3}-0.728x^{2}+19.322x+466.826$$

The material parameters $\left\{[\ln(C_{j}),m_{j}],j=1,...,4\right\}$ of the specimens *T*_1_–*T*_4_ were calculated as shown in Table 2, where the subscript *j* represents the specimen *T_j_*. The mean values ln(*C*) = −32, and *m* = 3.897 were chosen as the material constants *C* and *m* in the state equation.

The variance of the crack propagation uncertainty was approximated as $\sigma_{\omega }^{2}=0.166^{2}$ with the method mentioned above. The state equation is expressed as Equation (40):

(40) $$x_{k}=x_{k-1}+\exp(\omega )\times \exp(-32)\times {\left(0.016{x_{k-1}}^{3}-0.728{x_{k-1}}^{2}+19.322x_{k-1}+466.826\right)}^{3.897}\Delta N$$

**Figure 11:**
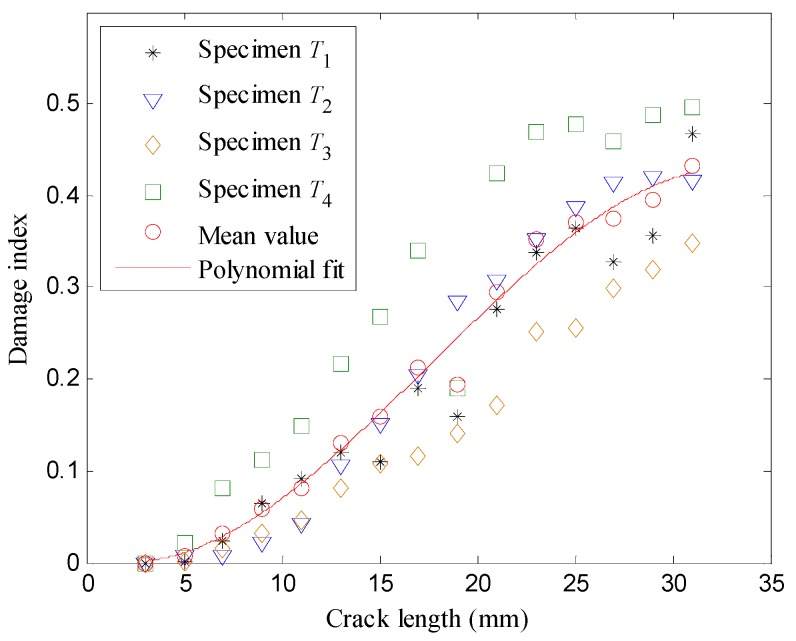
The relationship of damage index *versus* the crack length.

To obtain the measurement equation, the mean values of the damage indices were calculated by Equation (14) excluding the singular point. As shown in Figure 11, the 3rd order polynomial fit is applied for the mean values, which gives Equation (41):

(41) $$y=-2.962\times 10^{-5}\cdot x^{3}+0.00156x^{2}-0.00656x+0.00798$$

The variance of the measurement uncertainty was approximated from the experimental data as expressed in Equation (16). The maximum value ${\left(\sigma_{\nu }^{2}\right)}_{\max}=0.075^{2}$ was adopted in this paper. As a result, the measurement equation was obtained as Equation (42):

(42) $$y_{k}=-2.962\times 10^{-5}\cdot x_{k}^{3}+0.00156x_{k}^{2}-0.00656x_{k}+0.00798+\nu$$

where the variable ν subjects to normal distribution $N(0,0.075^{2})$.

#### 3.3.2. On-Line Crack Propagation Estimation and Prognosis

The particle number and the resampling threshold of the PF algorithm was set to $N_{s}=2000$, $N_{th}=0.8N_{s}$ respectively. The loading cycle step was chosen as Δ*N* = 50 cycles. The initial crack length is 3 mm. The $N_{s}$ random samples from the distribution $N(3,0.6^{2})$ were adopted as the initial particles to obtain a diverse particle set. When the crack length exceeds the predefined threshold $x_{\text{th}}=31\text{ mm}$, the structure is considered to be in a very critical state where the crack tip is close to the structure boundary, and is defined as structural failure.

**Figure 12:**
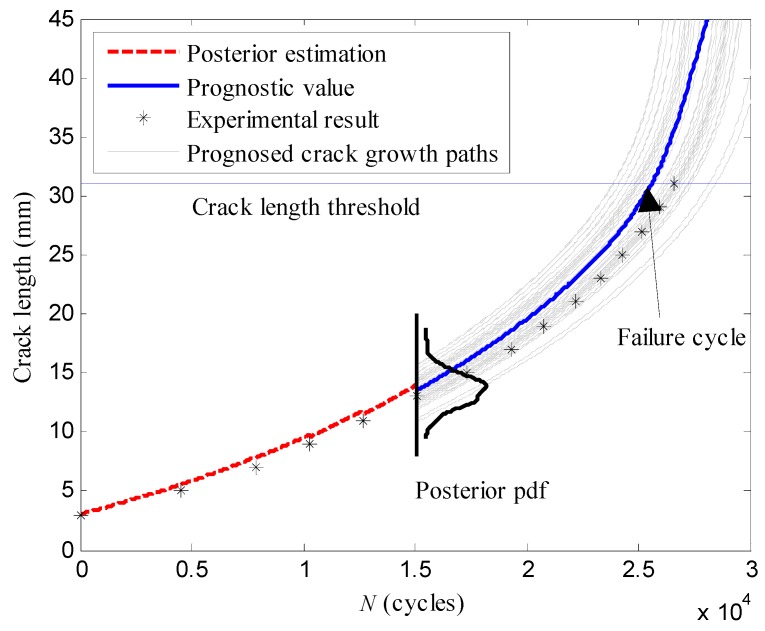
The estimation and prognosis procedures of the crack length with the LW-PF.

The specimen *T*_5_ was regarded as unknown and monitored by the active Lamb wave method. The damage indices extracted from the specimen *T*_5_ were used as the measurements of the actual crack lengths to estimate the posterior pdf of the crack length. The damage index was sequentially collected on-line. Once a new damage index was obtained, it was used for updating the weight. Figure 12 illustrates the posterior estimation and prognosis procedures with the proposed LW-PF based method. After the posterior pdf of the crack length is obtained using the available damage indices, the prognosis procedure is conducted based on the obtained posterior pdf. Each particle with the corresponding weight represents a possible crack propagation path. Taking all the possible paths, the prognostic values of the crack length in the future can be calculated, as well as the failure cycle.

**Figure 13:**
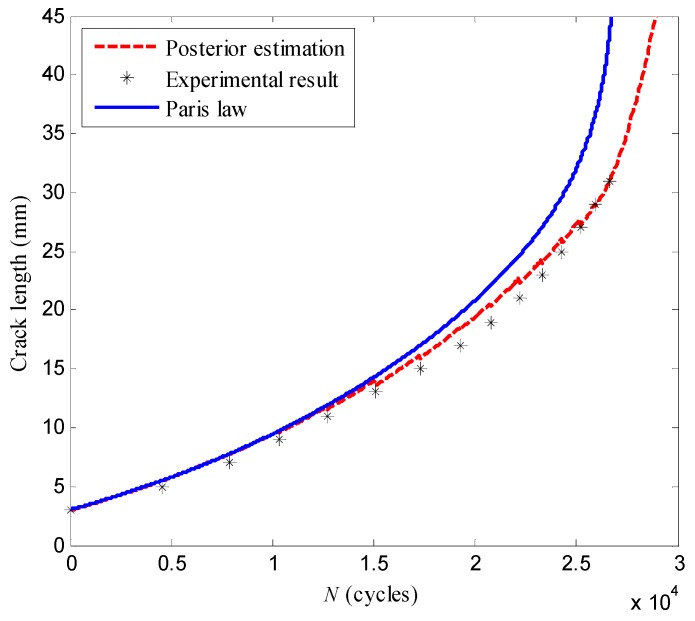
The crack propagation paths estimated with LW-PF and Paris’ Law.

As shown in Figure 13, the posterior estimations of the crack length considering all the damage indices are closer to the experiment results comparing with the results calculated directly by Paris’ law. This is because the LW-PF-based method takes advantage of the on-line monitored information with the crack propagation model. Table 3 gives the crack lengths estimated at *N* = 7848, *N* = 17,281, *N* = 23,286 cycles. As to the experimental results, it can be found that the errors of the LW-PF-based method are smaller than those obtained by Paris’ law.

**Figure 14:**
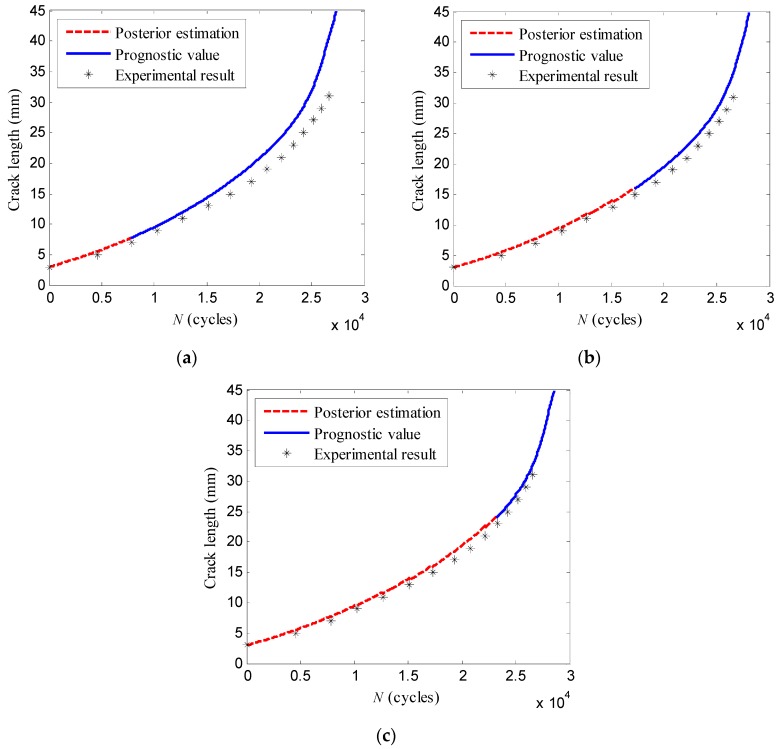
The crack propagation prognosis of the specimen *T*_5_ at: (**a**) *N* = 7848; (**b**) *N* = 17,281; (**c**) *N* = 23,286 cycles.

Moreover, the crack propagation paths predicted at loading cycles *N =* 7848, *N =* 17,281, *N =* 23,286 are shown in Figure 14. From these figures, it can be observed that the prognostic values of the crack lengths gradually approach the experimental results. Considering the prognostic value of the crack length at the future loading cycles *N =* 26,620, the results obtained at different cycles are shown in Table 4. The prognostic value of the proposed method becomes closer to the experimental value as more damage indices are available. Furthermore, the standard deviation of the prognostic pdf represented by the particles and corresponding weights decreases, which indicates the capability of uncertainty reduction of the proposed method.

#### 3.3.3. Failure Cycle Estimation of the Specimen *T*_5_

The crack length threshold is defined as $x_{\text{th}}=31\text{ mm}$. Once the crack length exceeds this threshold, the cracked structure is regarded as failed and the estimation of the failure cycle is obtained as expressed in Equation (37). The relative error of the failure cycle is specified as the percentage of the experimental failure cycle as shown in Equation (43):

(43) $$\text{Relative error}=\frac{\text{Estimated failure cycle-Experimental failure cycle}}{\text{Experimental failure cycle}}\times 100\%$$

As illustrated in Table 5, the estimations of the failure cycle at *N* = 7848, 17,281, 23,286 cycles are obtained, respectively. From the relative error of the estimated failure cycle, it can be found that the results have been improved by using the proposed method. As more damage indices become available, the prognostic estimation becomes more accurate, which shows the advantages of the proposed method.

### 3.4. Cross Validation Performed on the Specimen T_3_

For better validating the proposed method, a leave-one-out cross validation approach was employed by picking a different specimen as the target structure. In this validation, the specimen *T*_3_ was picked as the validation specimen instead, while the experimental data of the specimens *T*_1_, *T*_2_, *T*_4_, *T*_5_ was used for calculating the state space model parameters. By the same procedure, the state space model parameters of the specimen *T*_3_ were obtained as shown in Table 6.

**Figure 15:**
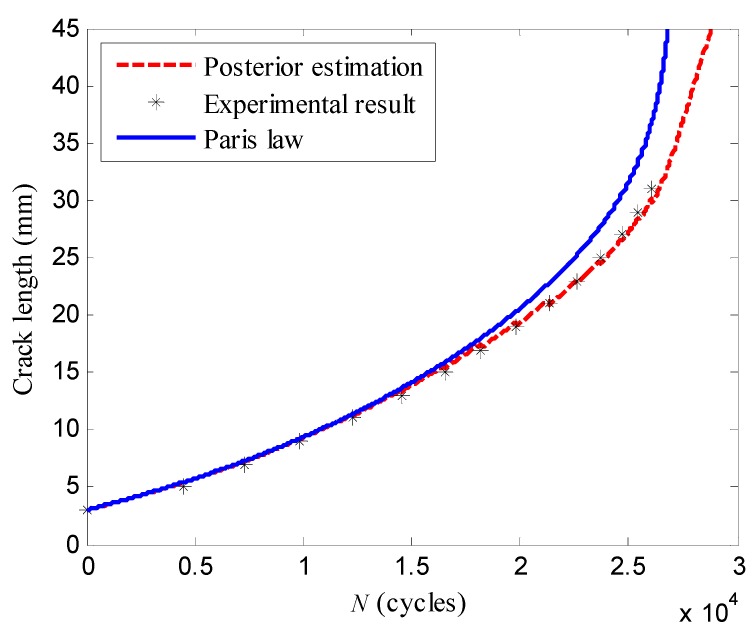
The posterior estimation of the specimen *T*_3_ considering all the damage indices.

The parameters of the PF algorithm, including the particle number, the resampling threshold, and the loading cycle step were set as the same values of the specimen *T*_5_. Figure 15 shows the posterior estimation of the crack propagation path considering all the damage indices. Similarly, the posterior estimations are closer to their experimental results comparing with the results using the Paris’ law. The estimations of the LW-PF based method at load cycles *N* = 7266, *N* = 16,514, *N* = 22,588 are shown in Table 7.

**Figure 16:**
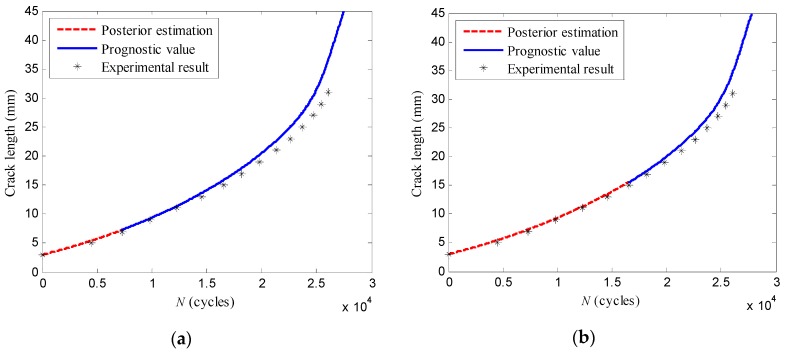

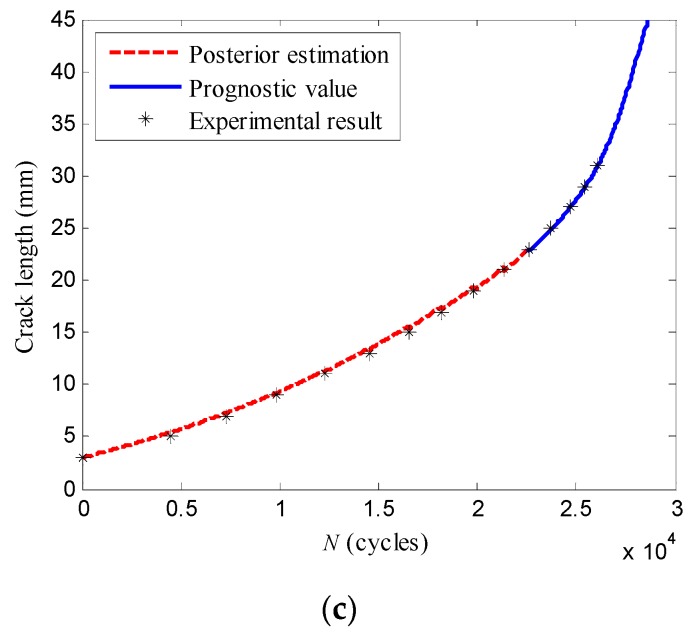
The crack propagation prognosis of the specimen *T*_3_ at: (**a**) *N* = 7266; (**b**) *N* = 16,514; (**c**) *N* = 22,588 cycles.

The crack propagation paths of the specimen *T*_3_ predicted at loading cycles *N* = 7266, *N* = 14,514, *N* = 22,588 are obtained as shown in Figure 16. In addition, Table 8 presents the prognostic crack length of the specimen *T*_3_ at future loading cycles *N* = 26,091. On the other hand, the estimations of the failure cycle obtained at loading cycles *N* = 7266, *N* = 16,514, *N* = 22,588 are shown in Table 9.

The validation experiment of the specimen *T*_3_ have also shown the advantage of the proposed method. By integrating the on-line monitored damage indices, the posterior estimations are more accurate than those obtained by Paris’ Law. Moreover, the prognostic crack propagation paths are improved as more damage indices are available, as well as the estimations of the failure cycle.

## 4. Conclusions

This paper proposes a LW-PF-based method for on-line crack propagation prognosis. In this method, the active Lamb wave-based method is employed for on-line crack monitoring. The cross-correlation damage indexes extracted from Lamb wave signals are used for measuring the actual crack length. To implement the PF algorithm, the parameters of the physical model-based state equation and the measurement equation are proposed to be predetermined through finite element analysis and data driven methods. The PF gives the probabilistic result for the crack propagation prognosis and the failure cycle estimation by integrating the on-line crack length measurements. The validation experiments performed on the hole-edge crack specimens have shown the capability of the proposed method.

## Figures and Tables

**Table 1:** The mechanical properties of LY12.

Material	Yield Strength (MPa)	Young Modulus (MPa)	Tensile Strength (MPa)
LY12	342	69,580	448

**Table 2:** The material parameters calculated from the specimens *T*_1_–*T*_4_.

Specimen	ln(*C*)	*m*
*T*_1_	−32.949	4.050
*T*_2_	−30.932	3.739
*T*_3_	−32.153	3.924
*T*_4_	−31.966	3.874
Mean	−32.000	3.897

**Table 3:** The posterior estimation of the crack length for the specimen *T*_5_.

Fatigue Load (Cycles)	*N* = 7848	*N* = 17,281	*N* = 23,286
Experimental result (mm)	7	15	23
Posterior estimation (mm)	7.71	15.92	23.99
Result of the Paris’ law (mm)	7.74	16.97	27
Error of the posterior estimation (mm)	0.71	0.92	0.99
Error of the Paris’ law (mm)	0.74	1.97	4

**Table 4:** The prognostic crack length of the specimen *T*_5_ for *N* *=* 26,620 cycles.

Fatigue Load (Cycles)	*N =* 7848	*N =* 17,281	*N =* 23,286	Paris’ Law	Experiment Result
Mean (mm)	40.2	35.1	32.7	43.1	31
Error of the mean (mm)	9.2	4.1	1.7	12.1	N/A
Standard deviation (mm)	5.99	5.16	3.27	N/A	N/A

**Table 5:** The estimation of the failure cycle for the specimen *T*_5_.

Fatigue Load (Cycles)	*N =* 7848	*N =* 17,281	*N =* 23,286	Paris’ Law	Eexperimental Data
Failure cycle (cycles)	25,116	25,903	26,357	23,840	26,620
Relative error	5.6%	2.6%	1.0%	10%	N/A

**Table 6:** The state space model parameters of the specimens *T*_3_.

Parameter	ln(*C*)	*m*	$\sigma_{\omega }^{2}$	Measurement Mapping	$\sigma_{v}^{2}$
Value	−32.405	3.960	0.169^2^	$y=-4.234\times 10^{-5}x^{3}+2.104\times 10^{-3}x^{2}-1.183\times 10^{-2}x+1.963\times 10^{-2}$	0.104^2^

**Table 7:** The posterior estimations of the crack length for the specimen *T*_3_.

Fatigue Load (Cycles)	*N* = 7266	*N* = 16,514	*N* = 22,588
Experimental result (mm)	7	15	23
Posterior estimation (mm)	7.28	15.71	23.51
Result of the Paris’ law (mm)	7.4	16.61	27.3
Error of the posterior estimation (mm)	0.28	0.71	0.51
Error of the Paris’ law (mm)	0.4	1.61	4.3

**Table 8:** The prognostic crack length of the specimen *T*_3_ for *N* = 26,091 cycles.

Fatigue Load (Cycles)	*N* = 7266	*N* = 16,514	*N* = 22,588	Paris’ Law	Experiment Result
Mean (mm)	36.7	34.9	31.5	36.9	31
Error of the mean (mm)	5.7	3.9	0.5	5.9	N/A
Standard deviation (mm)	7.4	6.2	4.0	N/A	N/A

**Table 9:** The estimation of the failure cycle for the specimen *T*_3_.

Fatigue Load (Cycles)	*N* = 7266	*N* = 16,514	*N* = 22,588	Paris Law	Eexperimental Data
Failure cycle (cycles)	24,851	25,160	25,936	24,820	26,091
Relative error	4.7%	3.5%	0.6%	8.8%	N/A
